# A New Method for Recognizing Protein Complexes Based on Protein Interaction Networks and GO Terms

**DOI:** 10.3389/fgene.2021.792265

**Published:** 2021-12-13

**Authors:** Xiaoting Wang, Nan Zhang, Yulan Zhao, Juan Wang

**Affiliations:** School of Computer Science, Inner Mongolia University, and with Ecological Big Data Engineering Research Center of the Ministry of Education, Hohhot, China

**Keywords:** protein interaction network, protein complex, GO terms, NNP, function of proteins

## Abstract

**Motivation:** A protein complex is the combination of proteins which interact with each other. Protein–protein interaction (PPI) networks are composed of multiple protein complexes. It is very difficult to recognize protein complexes from PPI data due to the noise of PPI.

**Results:** We proposed a new method, called Topology and Semantic Similarity Network (TSSN), based on topological structure characteristics and biological characteristics to construct the PPI. Experiments show that the TSSN can filter the noise of PPI data. We proposed a new algorithm, called Neighbor Nodes of Proteins (NNP), for recognizing protein complexes by considering their topology information. Experiments show that the algorithm can identify more protein complexes and more accurately. The recognition of protein complexes is vital in research on evolution analysis.

Availability and implementation: https://github.com/bioinformatical-code/NNP.

## Introduction

The recognition for protein complexes based on the PPI network has become one of the most important channels in current research. Detection of protein complexes from PPI networks is an important work in the understanding of biological processes. It is also of great significance for researching mechanisms and developing new drugs. Researchers have put forward a variety of effective methods to recognize protein complexes. The MCODE algorithm chooses a vertex with the maximum weight as the initial cluster, and then recursively searches for the vertices that meet a threshold value to add to the cluster (Bader and Hogue, 2003). The DPClus is a modified algorithm that chooses the vertices with high connectivity with the present cluster iteratively (Altaf-Ul-Amin et al., 2006). Jerarca uses the hierarchical cluster to partition the complexes based on the distance among proteins (Aldecoa and Marín, 2010). RNSC divides the complexes by means of a cost function (King et al., 2004). MCL (Enright et al., 2002) simulates network flow by constructing a similarity matrix, alternately performs expansion and inflation operations, and achieves clustering effect after multiple iterations. But the method is difficult to identify the complexes with little overlap. After that, an improved method was proposed which measured the reliability of PPI based on the annotations of protein function (Cho et al., 2007). SCI-BN and ClusterM combine topology of PPI and biological information of sequences to identify complexes (Qi et al., 2008; Wang et al., 2020).

Although these methods can effectively identify functional modules of proteins, they all ignore the internal structure of the modules. The basic structure of a protein complex is composed of the nucleus of a protein complex and all its subordinate proteins (Gavin et al., 2006). So, a protein complex can be regarded as a subgraph with a nucleus and its subordinate proteins for assisting the nucleus to play a specific role. COACH (Wu et al., 2009) and CORE (Leung et al., 2009) are proposed based on the idea. The F-MCL algorithm combines firefly algorithm and MCL (Lei et al., 2016). ClusterONE is a clustering algorithm guided by cohesion which can identify subgraphs of dense substructure (Nepusz et al., 2012). However, the cohesion formula may lead to deviation in the clustering process. EA (Halim et al., 2015) uses multi-population evolutionary algorithm to cluster the probability map. MNC is a novel clustering model based on multi networks which combines the shared clustering structure in PPI and domain–domain interaction (DDI) networks in order to improve the accuracy of identification (Ou-Yang et al., 2017). IdenPC-CAP recognizes protein complexes from the interaction networks consisting of RNA–RNA interactions, RNA–protein interactions, and PPIs (Wu et al., 2021). CSC uses both topological and biological characteristics to identify protein complexes (Liu et al., 2018; Sharma et al., 2018). DPCMNE detects protein complexes *via* multilevel network embedding (Meng et al., 2021). PC2P formalizes protein complexes as biclique spanned subgraphs and converts the problem of detecting protein complex to coherent partition (Omranian et al., 2021). A semi-supervised model based on non-negative matrix tri-factorization is also used to detect protein complex (Liu et al., 2021). In the FCAN-PCI, the semantic similarity of proteins and the topology of PPI network are integrated into a fuzzy clustering model (Pan et al., 2021). GECA proposes a model based on the gene expression and core-attachment (Noori et al., 2021). The idenPC-MIIP method modifies the weights of original network by defining mutually important neighbors on the weighted network and then identifies protein complexes using a greedy algorithm (Wu et al., 2021)

## Methods

For a PPI network *N*, TSSN computes the edge aggregation coefficient as the topology characteristics of *N*, makes use of the GO annotation as the biological characteristics of *N*, and then constructs a weighted network. NNP identifies protein complexes based on this weighted network.

### TSSN

A PPI network can be seen as an undirected graph *G*= (*V*, *E*), and each protein is a node in *V*. Two proteins interact with each other if and only if there is an edge between the two nodes representing two proteins. In order to describe the structural similarity among proteins in the PPI network, Jaccard coefficient between two nodes *u* and *v* in *G*= (*V*, *E*) is defined as follows:

$$J\left(u,v\right)=\frac{\left|N\left(u\right)\cap N\left(v\right)\right|}{\left|N\left(u\right)\cup N\left(v\right)\right|},$$ (1)

where *N*(*u*) [or *N*(*v*)] represents the set of all neighbor nodes of protein *u* (or *v*) in the network.

We adopted the simGIC method (Tian and Guo, 2017), which is an improved method from the GIC (Pesquita et al., 2007) to calculate semantic similarity between proteins. Assuming that proteins *u* and *v* are annotated by term sets *A*_*=*_{*T*_1_ *, T*_2_ *, ⋯, T*_*m*_} and *B*_*=*_ *{S*_*1*_ *, S*_*2*_ *, ⋯, S*_*n*_ *}* respectively, the semantic similarity between *u* and *v* is defined as follows:

$$se\left(u,v\right)=\frac{\sum_{T_{i}\in A\cap B}-\log p\left(T_{i}\right)}{\max\left\{IC\left(A\right),IC\left(B\right)\right\}},$$ (2)

Where *IC*(*A*) is the set of {−log(*T*_1_), −log(*T*_2_),…, −log(*T*_*m*_)}*,* and *p*(*T*_*i*_) represents the times that GO terms or single function of protein appear in the specified term data.

Here, the similarity between two proteins *u* and *v* is defined as the average between their topological similarity and semantic similarity, that is,

$$s\left(u,v\right)=\frac{\sum_{u_{1}\in N\left(u\right),v_{1}\in N\left(v\right)}\left(J\left(u_{1},v_{1}\right)+se\left(u_{1},v_{1}\right)\right)}{2},$$ (3)

where the value of *s*(*u,v*) is [0,1].

### NNP

Given a weighted network *G=* (*V, E, W*), where *V =* {*v*_1_ *, v*_2_ *, ⋯, v*_*m*_}, *E =* {*e*_1_ *, e*_2_ *, ⋯, e*_*n*_}, *W =* {*w*(*e*_1_)*, w*(*e*_2_)*, ⋯, w*(*e*_*n*_)}, and *w*(*e*_*i*_) represents the weight of the edge *e*_*i*_. The distance between the nodes *v*_*i*_ and *v*_*j*_ is the minimum among all lengths of paths. *V*_*j*_ is denoted as the set of nodes with the distance 2 between *v*_*j*_, which is referred to as the set of second-order neighbor nodes between *vj*. The network *G*_*j*_ *=* (*V*_*j*_ *, E*_*j*_ *, W*_*j*_) is derived by *V*_*j*_. The weighed degree of *v*_*j*_ in *G* is defined as follows:

$$WD\left(v_{j},G\right)=\sum_{i=1}^{n}w\left(v_{j},v_{i}\right),$$ (4)

where (*v*_*j*_ *, v*_*i*_) ∈ *E* and *w*(*v*_*j*_ *, v*_*i*_) indicates the weight of the edge between node *j* and node *i*. The average weighted degree of *v*_*j*_ in *G* is computed by the following equation:

$$AWD\left(v_{j},G\right)=\sum_{i=1}^{n}w\left(v_{j},v_{i}\right)/|V|.$$ (5)

The weighted neighbor ratio is defined as follows:

$$WN\left(v_{j},G\right)=\frac{WD\left(v_{j},G\right)}{WD\left(v_{j},G\right)+WD\left(v_{j},G_{j}\right)}.$$ (6)

In order to assess complexes, we compute the tightness degree of a complex *G=* (*V, E, W*) as follows:

$$WDt\left(G\right)=2\sum_{i=1}^{n}w\left(e_{i}\right)/\left(\left|V\right|\times \left(\left|V\right|-1\right)\right).$$ (7)

For two complexes C1 and C2, the overlap ratio (OL) between them is defined as follows:

$$OL\left(C_{1},C_{2}\right)=\frac{{\left|C_{1}\cap C_{2}\right|}^{2}}{\left|C_{1}\right|\cdot \left|C_{2}\right|}.$$ (8)

**FIGURE 1:**
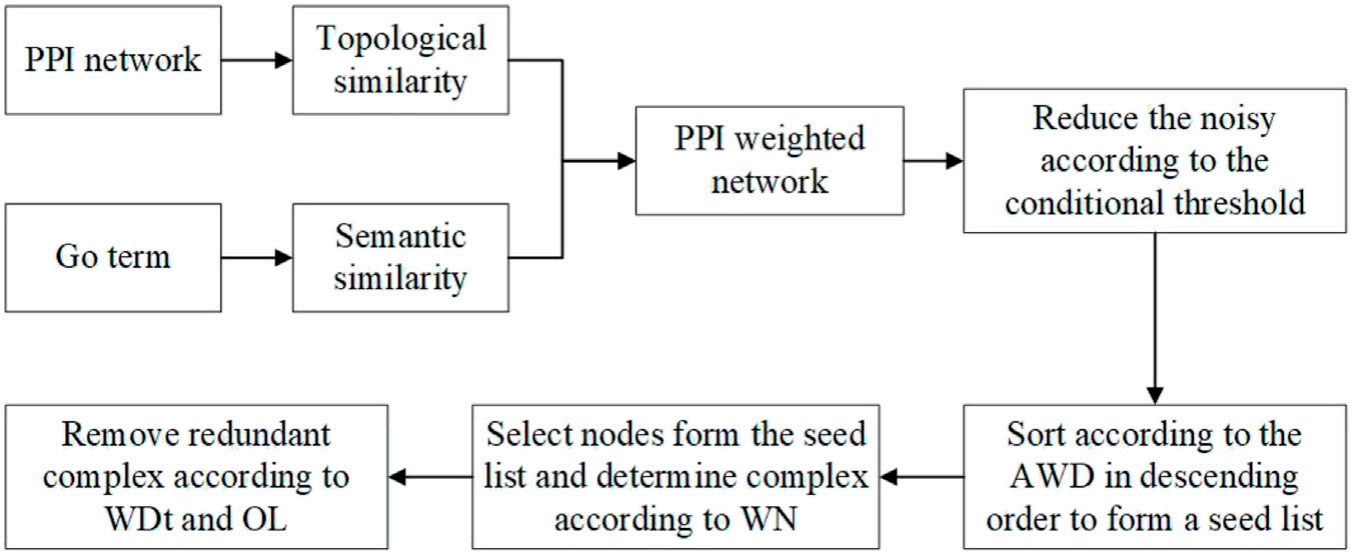
Workflow of the NNP.

NNP identifies complexes by four main steps. First, the NNP uses the TSSN method to compute the similarity among proteins, and then builds a PPI weighted network and neighbor networks. Second, it calculates a conditional threshold in order to reduce the noise, and then the network is transformed into a matrix, which is arranged in descending order according to the average weighted degree (AWD) of nodes to form a seed list. Third, it selects nodes from the seed list iteratively as the initial complex to cluster, and then removes or retains the node according to the weighted neighbor ratio (WN) until all nodes list are solved. Finally, it calculates the OL among protein complexes and judges whether the complexes are retained or discarded through the network tightness (WDt). Finally, the complex set was obtained. Figure 1 shows the workflow of NNP. The pseudo code can be seen in the Algorithm.

## Results and Discussion

In order to assess the TSSN method, we compare the protein complexes identified by three classical methods, that is, ClusterONE, MCODE, and MCL, respectively, based on the PPI networks with the weight computed by TSSN and the PPI networks without weight. We compare the results of protein complexes predicted by CFinder, ClusterONE, MCODE, MCL, EA, and NNP methods.

### Datasets

In all experiments, we use the PPI data of yeast downloaded from the DIP database (https://dip.doe-mbi.ucla.edu/dip/Download.cgi?SM=7&TX=4932), version 20170205. In order to reduce the noise of data, we delete the repeated interactions and the circle of a node to itself. Then the PPI network contains 5,115 nodes and 22,552 edges. GO annotations and ontology data of yeast are downloaded from the website (http://www.geneontology.org/).

### Reference Sets

Here, two standard sets, namely, CYC2008 (Pu et al., 2009) and NewMIPS (Friedel et al., 2008), are used in the experiments, where CYC2008 is downloaded from (http://wodaklab.org/cyc2008/downloads). These data are predicted by biological methods, including 408 complexes and 1,628 proteins. The NewMIPS is a set of protein complexes, including 428 complexes and 1,171 proteins.

### Metrics

For a prediction algorithm, its effectiveness is measured by four indexes: recall, precision, F1, and overlap ratio. The recall value *R* is the ratio of the number of complexes which are identified by methods and matched with the complexes in the standard set to the number of complexes in the standard set; the precision value *P* is the ratio of the number of complexes which are identified by methods and matched with the complexes in the standard set to the number of all complexes identified by the algorithm. F1 is the harmonic average of *P* and *R*, that is,

$$F1=\frac{2\times R\times P}{R+P}.$$ (9)

To judge the biological significance of complexes, a functional enrichment analysis is used to analyze the gene annotation information in the GO database, that is, *p*-value. The calculation method is given as follows:

$$p-value=1-\sum_{i=0}^{m-1}\frac{\left(\begin{array}{c} \left|F\right|\\ i \end{array}\right)\left(\begin{array}{c} \left|V|-|F\right|\\ |C|-i \end{array}\right)}{\left(\begin{array}{c} \left|V\right|\\ \left|C\right| \end{array}\right)},$$ (10)

where *m* is the number of identified complexes that are the same as those in the standard data set, *F* the complexes in the standard data set, *V* the number of proteins contained in the PPI network, and *C* the number of identified complexes. Here, if *p-value* is less than 0.01, the complex is regarded with biological significance.

## Results

In all recorded experimental results, we use CYC2008 as the standard set and set the threshold of OL as 0.2. OL represents the overlap rate between the two complexes. The value of OL being 0.2 indicates that the identified complex is considered correct when the OL with the standard complex reaches 0.2.

**Algorithm:** detecting protein complexes.

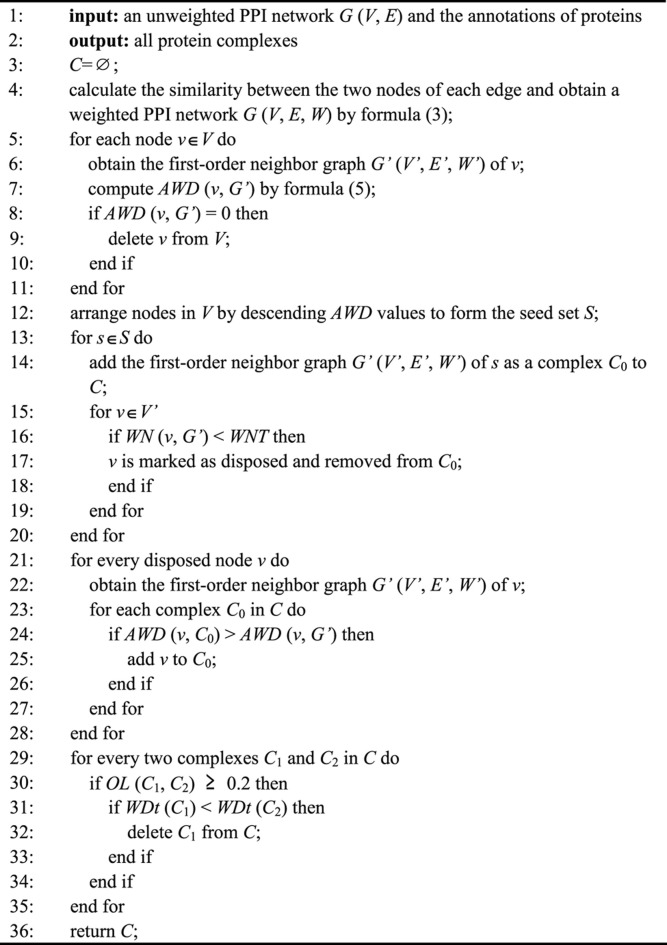

Table 1 shows the results. For each method in Table 1, u represents the methods that are used to identify the complexes from the unweighted networks and T represents the methods that are used to identify the complexes from the weighted networks computed by the TSSN. From Table 1, we can see that the precision values for the weighted networks computed by the TSSN method are higher than those for the unweighted networks. So the TSSN method is efficient for computing the weigh values of networks.

**TABLE 1:** Results of methods are used in the unweighted networks and weighted networks computed by the TSSN.

Metrics Method	*R*	*P*	F1
ClusterOne-u	0.32	0.415	0.361
ClusterOne-T	**0.34**	**0.43**	**0.38**
MCODE-u	0.21	0.49	0.294
MCODE-T	**0.23**	**0.51**	**0.317**
MCL-u	0.58	0.21	0.308
MCL-T	**0.605**	**0.228**	**0.331**

Bold values represents the experimental results on ClusterOne, MCode and MCL weighted by the TSSN method.

The precision results of the NNP algorithm depend on the thresholds of weighted neighbor ratio (WNT). Table 2 shows that F1 values gradually increase with the increase in *t* values if the thresholds of WNT is (0,0.2), and F1 gradually decreases as a whole if the t values of WNT continue to increase from 0.2. So F1 can reach the maximum 0.42 if values of WNT are (0.2, 0.25). Table 3 shows the precision values of NNP on different thresholds of WNT. When the WNT value is 0.22, the precision is 0.5, which is slightly higher than the other five values. Therefore, it is reasonable for the NNP algorithm to set the threshold of the WNT as 0.22.

**TABLE 2:** F1 values of NNP on different thresholds of WNT.

*t*	0	0.1	0.2	0.3	0.4	0.5	0.6	0.7	0.8	0.9	1
F1	0.4	0.41	**0.42**	0.41	0.4	0.39	0.395	0.37	0.3	0.2	0.13

Bold values shows that when the threshold t is 0.2, the value of F1 reaches a maximum of 0.42.

**TABLE 3:** Precision values of NNP on different thresholds of WNT.

t	0.2	0.21	0.22	0.23	0.24	0.25
Precision	0.491	0.492	**0.5**	0.495	0.493	0.493

Bold values shows that when the threshold t is 0.5, the precision value reaches the maximum 0.5.

Table 4 lists the comparison of the cluster information identified by the six algorithms compared with CYC2008. CYC2008 is selected as the benchmark, and its average size is 4.71; the closer the average size of the cluster identified by a method is to 4.71, the more accurate the method is. Among the six algorithms, the average size of clusters identified by the NNP is 4.54, which is closest to the size of clusters in the standard data. So the recognition result of NNP has high theoretical reliability.

**TABLE 4:** Each algorithm identifies the cluster information.

No.	Algorithm	Number	Average	Coverage
1	CYC2008	408	4.71	1,628
2	CFinder	178	11.31	2,147
3	ClusterONE	413	5	1898
4	MCODE	110	6.5	1,299
5	NNP	538	4.54	1937
6	MCL	623	6.57	4096
7	EA	398	13.5	2,661
8	PC2P	434	4.50	1953

Table 5 shows the results identified by the CFinder, ClusterONE, MCODE, MCL, EA, NNP, and PC2P methods for three complexes randomly selected from DIP. CFI is the mRNA cleavage factor complex with size 5; NEC is the nuclear exosome complex with size 12, and DRC is the DNA-directed RNA polymerase II complex. The table shows that six methods recognize the same proteins as the CYC2008 for the CFI, that is, OL 100%, OL of NNP, and MCL is both 100% for NEC. The OL of PC2P is 83.3%. The OL of EA and that of MCODE are the same, which is 91.7%, ranking second. There is one missed protein: YHR081W. CFinder has two missed proteins and the OL is 84%. The OL of PC2P is 83.3%. So, the accuracy of ClusterONE is low. For DRC, the performance of NNP and ClusterONE is better, while the OL value of EA is 83.3%. There are many omissive and wrong proteins detected by CFinder, MCODE, MCL, and PC2P. The OL of CFinder is 56.3%. The OL of PC2P is only 53.3%.

**TABLE 5:** Three complexes identified by methods were analyzed from the DIP.

Algorithm Protein complex	CFinder (%)	Cluster -ONE	MCODE (%)	NNP (%)	MCL (%)	EA (%)	PC2P (%)
CFI	100	100%	100	100	100	100	83.3
NEC	83.3	64.1%	91.7	100	100	91.7	83.3
DRC	56.3	100%	61.4	91.7	67.5	83.3	53.3

Table 6 shows the results of six methods. In terms of precision, the value of CFinder is lowest, which is only 26.98%, and the value of NNP is largest compared with other algorithms, reaching 51.07%. The precision of MCODE lists second, reaching 50.1%. Although the precision of MCODE is high, the recall is low, which leads to the low F1 value. From the table, it is obvious that the F1 of NNP is max among all other methods. So NNP has better accuracy in identifying protein complexes than other methods.

**TABLE 6:** Results of protein complexes recognized by algorithms.

Metrics method	*R*	*P*	F1
CFinder	0.3408	0.2698	0.3012
ClusterONE	0.4068	0.3554	0.3794
MCODE	0.2293	0.501	0.3146
NNP	**0.3515**	**0.5107**	**0.4164**
MCL	0.3326	0.4093	0.367
EA	0.34	0.383	0.3602
PC2P	0.4340	0.1935	0.2677

Bold values show that the experimental results of the NNP method are optimal.

Table 7 lists the number of protein complexes identified by CFinder, ClusterONE, MCODE, MCL, EA, NNP, and PC2P from DIP data set, matched with CYC2008. As shown in Table 7, the protein complexes identified by NNP based on the DIP data set are perfectly matched with 17 protein complexes. The MCODE only has six complexes perfectly matched with the standard set. The PC2P has no perfectly matched complex with the standard set. Therefore, compared with other algorithms, the NNP algorithm can accurately and perfectly match more protein complexes on the DIP data set.

**TABLE 7:** Numbers of protein complexes perfectly matched by each algorithm for DIP data set.

Algorithm	Perfect matching
CFinder	11
ClusterONE	10
MCODE	6
NNP	**17**
MCL	15
EA	14
PC2P	0

Bold values show that the experimental results of the NNP method are optimal.

Table 8 lists some protein complexes with low *p-values* identified by the NNP algorithm on the DIP, which can show that the protein complexes identified by the NNP algorithm have significant biological significance. Table 9 lists three protein complexes perfectly matched with DIP and NewMIPS identified by the NNP method.

**TABLE 8:** Protein complexes with lower *p*-value identified by the algorithm on the DIP.

GO term	OL (%)	*p*-value
mRNA processing	96	1.54E-36
Small nuclear ribonucleo protein complex	86.1	2.73E-58
mRNA splicing, *via* spliceosome	95.7	4.48E-38
Transferase activity, transferring glycosyl groups	89.59	1.81E-76
Ribosomal small subunit biogenesis	88.2	2.45E-48
Transporter activity	94.38	6.84E-100

**TABLE 9:** Algorithm perfectly matches the protein complex on the DIP.

GO term	OL (%)	*p*-value
mRNA metabolic process	100	7.37E-27
Anaphase-promoting complex–dependent catabolic process	100	4.68E-24
Polyadenylation-dependent snoRNA 3′-end processing	100	1.45E-32

## Conclusion

Considering the topological structure of the PPI network, it introduces the gene ontology in biological information. We propose the methods for computing weight of protein interaction network and the recognizing of protein complexes on the weighted network. By comparing with other algorithms, the TSSN method based on topological features and GO term similarity can filter the noise, which can reduce the impact of noise data. The NNP algorithm can identify the protein complexes. The experimental results show that the NNP is superior to other classical algorithms.

In the future, we will adopt new technologies to detect false-positive edges and predict false-negative edges in the PPI network, thus improving the quality of the PPI network. Machine learning methods will be used to detect protein complexes based on their biological characteristics. Finally, since static PPI networks only contain the interaction between proteins and cannot reflect the dynamic characteristics of proteins interactions over time, we will study how to build a dynamic PPI network and identify protein complexes in the dynamic network.

## Data Availability

The original contributions presented in the study are included in the article/Supplementary Material; further inquiries can be directed to the corresponding author.
